# Erythroferrone is not required for the glucoregulatory and hematologic effects of chronic erythropoietin treatment in mice

**DOI:** 10.14814/phy2.13890

**Published:** 2018-10-12

**Authors:** Richard Coffey, Ugo Sardo, Léon Kautz, Victoria Gabayan, Elizabeta Nemeth, Tomas Ganz

**Affiliations:** ^1^ Department of Medicine David Geffen School of Medicine University of California Los Angeles California; ^2^ IRSD Université de Toulouse INSERM U1220 INRA U1416 ENVT UPS Toulouse France

**Keywords:** Erythroferrone, erythropoietin, glucose, hepcidin, myonectin

## Abstract

Erythropoietin (EPO) acts on erythroid progenitor cells to promote their survival and differentiation to mature erythrocytes. Along with this canonical role, EPO is also reported to modulate energy metabolism, resulting in improved glucose tolerance and insulin sensitivity. EPO also stimulates the production of the hormone erythroferrone (ERFE) which acts to suppress hepcidin production, thus increasing dietary iron absorption and mobilizing stored iron for use in erythropoiesis. ERFE (initially termed myonectin) was also reported have an effect on systemic lipid metabolism by promoting the clearance of nonesterifed fatty acids (NEFA) from circulation. As increased levels of circulating NEFA blunt insulin sensitivity and impair glucose tolerance, ERFE‐induced clearance of NEFA after EPO administration would have a beneficial effect on glucose metabolism. The aim of this study was to determine if the known metabolic effect of EPO treatment on glucose homeostasis is mediated by ERFE, produced in response to EPO. Mice lacking *Erfe* did not differ from wild‐type mice in blood lipid parameters, blood glucose, and glucose or insulin tolerance at baseline or after chronic EPO treatment. Additionally, hepcidin suppression and the response of erythrocyte parameters to chronic EPO treatment were unaffected by the absence of *Erfe*. These findings suggest that the known beneficial effects of EPO on glucose metabolism are not attributable to an accompanying increase in ERFE production, and that *Erfe* is dispensable for normal glucose homeostasis. Furthermore, our data indicate that ERFE‐independent mechanisms can suppress hepcidin in response to chronically elevated EPO levels.

## Introduction

In vertebrates, most of the body's iron is contained within hemoglobin in erythrocytes, where it binds oxygen for transport to tissues. Steady‐state erythropoiesis consumes more than 80% of circulating iron (Finch et al. 1970) to replace senescent erythrocytes that are removed from circulation by macrophages of the reticuloendothelial system (Knutson and Wessling‐Resnick 2003). The production of new erythrocytes can be substantially increased in response to erythropoietic stress after adverse events such as blood loss or hemolysis (Coleman et al. 1953; Hillman and Henderson 1969), which further increase iron requirements. Inadequate iron delivery to the erythron impairs erythropoiesis and can manifest as hypochromic and microcytic anemia (Barton and Bottomley 2000). Erythropoiesis is regulated by erythropoietin (EPO), a glycoprotein hormone produced by the kidney in response to hypoxia. EPO increases erythrocyte production by preventing the programmed cell death of erythroid precursors and promoting their maturation (Koury and Bondurant 1988, 1990). Beyond this canonical effect of EPO in regulating erythropoiesis, EPO also indirectly supports erythropoiesis by increasing the production of the hormone erythroferrone (ERFE). ERFE, a member of the C1q/TNF‐related protein family, is encoded by the gene *ERFE*, formerly named *FAM132B*, and is secreted into circulation by erythroblasts in response to elevated plasma EPO concentrations (Kautz et al. 2014). ERFE functions to enhance iron availability to match increased erythropoiesis: ERFE acts directly on hepatocytes to suppress the production of hepcidin (Kautz et al. 2014), the master regulator of organismal iron homeostasis that inhibits iron absorption and mobilization from stores (Nemeth et al. 2004). Mice lacking *Erfe* fail to acutely suppress hepcidin after bleeding and take longer to recover from anemia (Kautz et al. 2014).

In addition to promoting erythropoiesis, EPO signaling also elicits systemic metabolic effects. Treatment with EPO increases insulin sensitivity and glucose tolerance in humans (Allegra et al. 1996; Mak 1998) and mice (Katz et al. 2010; Foskett et al. 2011; Alnaeeli et al. 2014). The protein encoded by the *ERFE* gene was initially termed myonectin, a myokine secreted by skeletal muscle and linked to lipid metabolism and nutrient sensing (Seldin et al. 2012, 2013). In those studies, injection of recombinant ERFE reduced serum nonesterified fatty acid (NEFA) levels in mice, and in vitro ERFE treatment increased NEFA uptake by hepatocyte‐ and adipocyte‐derived cell lines (Seldin et al. 2012). Elevated NEFA levels are known to decrease insulin‐stimulated glucose uptake (Boden et al. 1994; Boden and Chen 1995) so ERFE‐mediated reduction of circulating NEFA levels would be expected to increase glucose tolerance and insulin sensitivity (Santomauro et al. 1999). We therefore surmised that some of the metabolic effects of EPO could be mediated by its ability to induce ERFE secretion by erythroblasts.

In this study, we used *Erfe* knockout mice (*Erfe* ^*−/−*^) to determine whether ERFE at physiologic concentrations modulates circulating NEFA levels, and to what extent increased glucose tolerance and insulin sensitivity after prolonged treatment with EPO is attributable to its stimulation of ERFE production. Our findings indicate that, at physiologic concentrations, ERFE does not alter plasma lipid homeostasis and that increased ERFE production does not mediate the effect of EPO on blood glucose homeostasis. Additionally, we report that the absence of ERFE does not measurably restrict erythropoiesis during chronic EPO treatment.

## Materials and Methods

### Experimental animals

*Erfe* ^+/−^ mice on a C57BL/6J background, described previously (Kautz et al. 2015), were bred to generate littermate *Erfe* ^*−/−*^ mice and wild‐type (WT) controls used for metabolic testing, serum NEFA measurements, complete blood counts (CBC), and iron parameter analysis. For chronic treatment experiments, mice were injected intraperitoneally with either 200 U recombinant mouse EPO (Biolegend) or sterile saline 3x per wk, on Monday, Wednesday, and Friday, beginning at 6 weeks and ending at 8 weeks of age. The final injection was performed 15 h prior to termination by isoflurane inhalation, and mice were fasted during those 15 h. Because metabolic testing requires frequent blood sampling, separate groups of mice were used for metabolic testing from those that were used for the analysis of serum NEFA, CBC, and iron parameters.

WT C57BL/6J mice used in acute EPO treatment experiments were obtained from the Jackson Laboratory at 5 weeks of age and housed at UCLA until testing. At 8 weeks of age, mice were injected intraperitoneally with either a single dose of 200 U recombinant mouse EPO or sterile saline 15 h prior to sacrifice, and were also fasted during this time. Mice used in these experiments were maintained on a low‐fat, low‐sucrose (3.25%) natural ingredient diet containing approximately 185 ppm iron (Labdiet, #5053) and housed in a specific pathogen‐free barrier facility at UCLA.

*Erfe* ^−/−^ mice used in the characterization of blood lipid and blood glucose parameters at 10 and 18 weeks of age were generated by breeding *Erfe* ^−/−^ mice on a C57BL/6J background. WT C57BL/6J mice used as controls were obtained from Janvier Laboratories at 5 weeks of age. Mice were fed a low‐fat, low‐sucrose (7%) purified diet (Ssniff Spezialdiäten GmbH, #E157453) and sacrificed by retro‐orbital exsanguination after either unrestricted access to food (the 10‐week age group) or after a 16 h fast (the 18‐week age group). Mice used in these experiments were housed in a specific pathogen‐free barrier facility in the animal facilities of INSERM US006. All experimental protocols involving mice reported in this manuscript were carried out with approval from the University of California, Los Angeles and the Université de Toulouse.

### Glucose and insulin tolerance testing

At 8 weeks of age, mice were injected with either EPO or sterile saline and fasted 15 h overnight prior to glucose tolerance testing. Mice were injected intraperitoneally with 1.5 g/kg bodyweight glucose diluted in sterile saline, and blood glucose levels were measured immediately prior to injection and at 15, 30, 60, and 120 min. Insulin tolerance testing (ITT) was performed 2 days after glucose tolerance testing. Mice were injected with an additional dose of either EPO or sterile saline 21 h prior to, and fasted for 6 h prior to, the start of ITT. Mice were injected intraperitoneally with 0.75 U/kg human insulin (Novolin R, Novo Nordisk) diluted in sterile saline and blood glucose levels were measured immediately prior to injection, and at 15, 30, 45, and 60 min. Glucose measurements were obtained by using an AlphaTRAK 2 hand‐held glucometer (Zoetis). During both glucose and insulin tolerance testing, blood was obtained from the distal tail tip.

### Iron parameter analysis and complete blood counts

Liver nonheme iron levels were measured, following acid digestion, by a colorimetric assay according to the manufacturer's protocol (Sekisui Diagnostics). Livers were homogenized prior to sampling for nonheme iron analysis, to prevent variability resulting from differences in the regional distribution of iron. Complete blood counts were obtained using a HemaVet blood analyzer (Drew Scientific).

### Quantification of serum ERFE, hepcidin, and blood lipid parameters

Serum ERFE levels were determined as described previously (Kautz et al. 2015) using antibodies developed by Silarus Therapeutics, La Jolla, CA. Serum hepcidin concentrations were determined by ELISA as previously detailed (Kautz et al. 2014) using anti‐hepcidin antibodies developed by Amgen. Serum NEFA levels were measured by using the HR Series NEFA‐HR2 kit (Wako Diagnostics). Blood lipid and glucose parameters were analyzed in mice at 10 and 18 weeks of age by using an ABX Pentra 400 clinical chemistry analyzer (Horiba Medical).

### RNA isolation and gene expression analysis

Total RNA was isolated from tissues by using Trizol (ThermoFisher Scientific). cDNA was synthesized by using the iScript cDNA Synthesis Kit (Bio‐Rad) according to the manufacturer's protocol. Relative mRNA levels for genes of interest were quantified by using qRT‐PCR and SsoAdvanced Universal SYBR Green Supermix (Bio‐rad) run on a CFX96 Real‐Time PCR Detection System. The following primer sequences were designed to detect all known transcript isoforms. m*Hprt (*hypoxanthine guanine phosphoribosyl transferase):,F; *CTG‐GTT‐AAG‐CAG‐TAC‐AGC‐CCC‐AA* R; *CAG‐GAG‐GTC‐CTT‐TTC‐ACC‐AGC*, m*Erfe*: F; *ATG‐GGG‐CTG‐GAG‐AAC‐AGC* R; *TGG‐CAT‐TGT‐CCA‐AGA‐AGA‐CA*.

### Statistical analysis

Statistical analysis was performed by using the SigmaPlot 12.5 package (Systat Software). Groups of mice were compared with other groups of the same sex, to eliminate variability resulting from sex differences in parameters related to iron or glucose homeostasis (Macotela et al. 2009; McLachlan et al. 2017). Group means were compared by using the student's *t*‐test, one‐way ANOVA, two‐way repeated measures ANOVA, or three‐way ANOVA where indicated. In response to differences between group means, Tukey's (for one and three‐way ANOVA) or Holm–Sidak (for two‐way repeated measures ANOVA) multiple comparisons testing was performed to determine which groups differed significantly. Differences between groups were considered significant at a *P* value of < 0.05.

## Results

### The effect of ERFE on serum lipid levels

**Figure 1:**
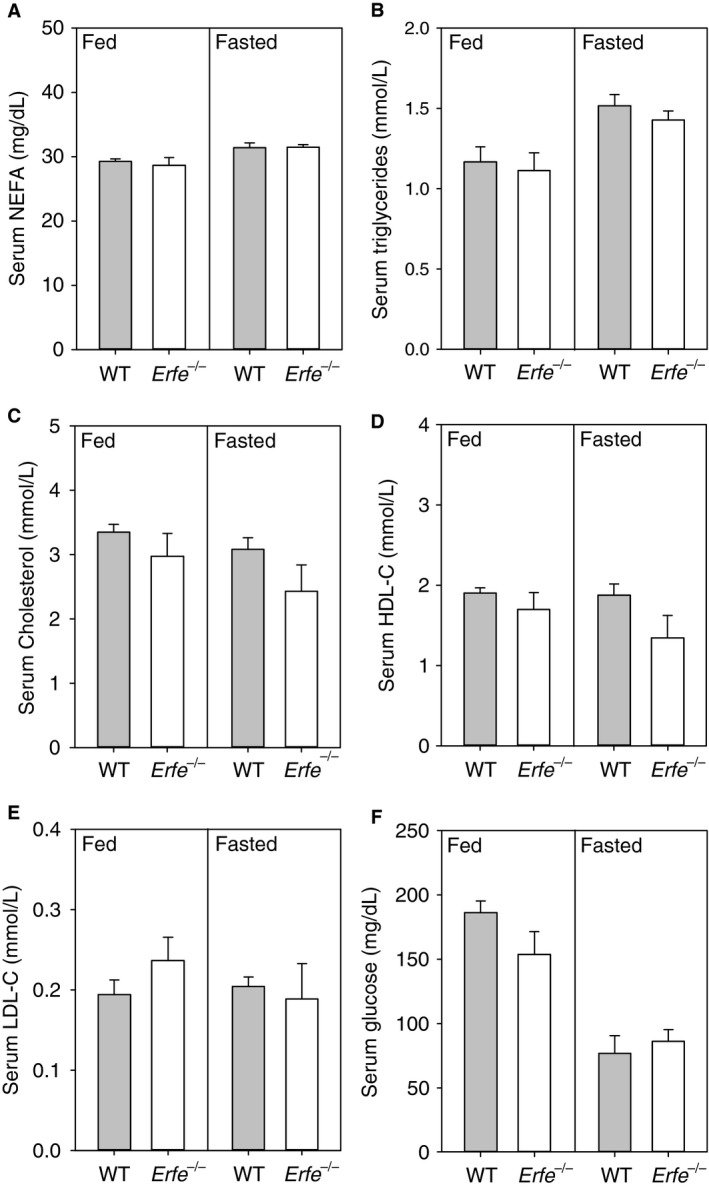
*Erfe* ^*−/−*^ mice have normal blood lipid levels during both fed and fasted conditions. Serum NEFA (A), triglyceride (B), total cholesterol (C), HDL‐C (D), LDL‐C (E), and serum glucose levels (F) in male WT and *Erfe* ^*−/−*^ knockout mice that had access to food or were fasted for 16 h prior to sacrifice. Parameters were measured at 10 weeks of age in fed mice and at 18 weeks in fasted mice (*n* = 8–10 per group). Group means between WT and Erfe‐/‐ mice were independently compared under either fed or fasted conditions by using the student's *t*‐test. Different superscripts indicate statistical significance (*P *<* *0.05). Values are presented as group means ± SEM.

To determine whether the absence of ERFE results in altered serum lipid homeostasis under baseline physiologic conditions, we measured serum NEFA, triglyceride, total cholesterol, HDL‐C, and LDL‐C levels in male WT and *Erfe* ^*−/−*^ mice under both fed and fasted conditions (Fig. 1A–E). We detected no statistically significant difference in blood lipid parameters between genotypes during either nutritional states. Blood glucose levels were also not different between WT and *Erfe* ^*−/−*^ mice during either fed or fasted conditions (Fig. 1F).

**Figure 2:**
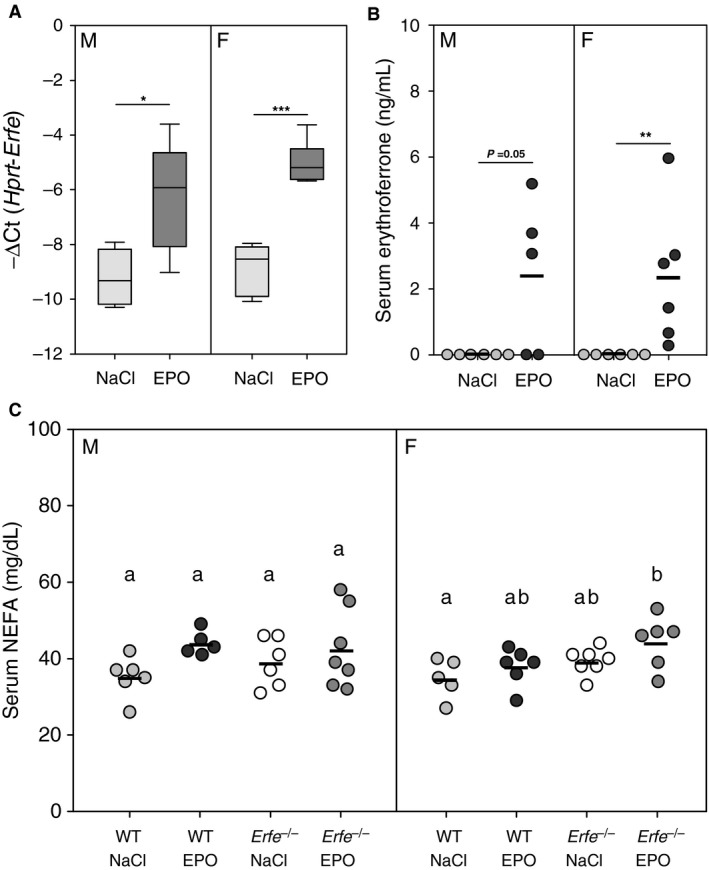
Serum levels of nonesterified fatty acids are not affected by physiologic concentrations of ERFE. Bone marrow mRNA expression of *Erfe* (A) and serum ERFE levels (B) in WT mice after chronic treatment with either EPO or saline (NaCl) (*n* = 5–6 per group for each sex). (C) Serum nonesterified fatty acid (NEFA) concentrations in WT and *Erfe* ^*−/−*^ mice after chronic treatment with either EPO or saline (NaCl) (*n* = 5–7 per group for each sex). In panels B and C data are presented as individual values from experimental animals with a line indicating the group mean. Group means between mice of the same sex were compared by using either the student's t‐test (A and B) or one‐way ANOVA (C). Asterisks indicate a statistically significant difference between groups as determined by *t*‐test (*P <* *0.05, **P <* *0.01, ***P <* *0.001) and means without a common alphabetical superscript differ significantly as determined by one‐way ANOVA (*P *<* *0.05). Data are shown as the mean ± SEM or as individual data points.

Circulating ERFE levels are low under baseline conditions and increase in response to erythropoietic stimuli (Kautz et al. 2015). Therefore, we chronically treated WT and *Erfe* ^*−/−*^ mice with either high‐dose EPO or saline on alternating days for 2 weeks to determine if increased levels of ERFE alter serum lipid homeostasis. We focused on NEFA levels as treatment with ERFE has been reported to modulate serum NEFA concentrations (Seldin et al. 2012). In response to chronic treatment with EPO, bone marrow mRNA expression of *Erfe* was elevated compared with that of saline‐treated controls and serum ERFE levels increased from below the threshold of detection (Fig. 2A and B). Despite the upregulation of ERFE expression by EPO treatment, serum NEFA levels were not different between WT and *Erfe* ^*−/−*^ mice during EPO‐stimulated conditions (Fig. 2C).

**Figure 3:**
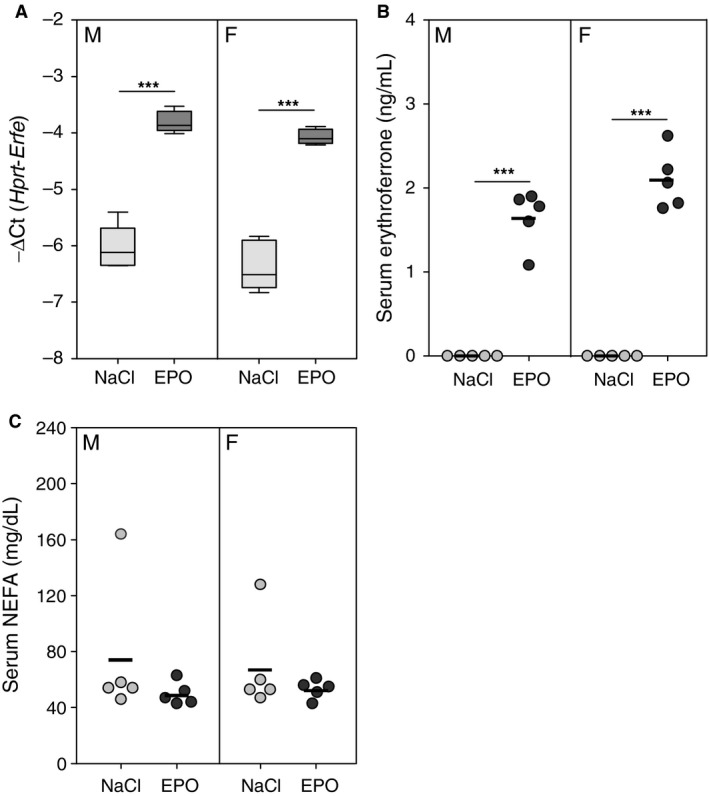
Serum levels of nonesterified fatty acids are not affected by acute EPO treatment. Bone marrow mRNA expression of *Erfe* (A), serum erythroferrone levels (B), and serum nonesterified fatty acid (NEFA) concentrations in wild‐type mice 15 h after treatment with a single dose of either 200 U EPO or saline (NaCl). In panels B and C, data are presented as individual values from experimental animals with a line indicating the group mean. Group means between mice of the same sex were compared by using the student's *t*‐test. Asterisks indicate a statistically significant difference between groups (***P <* *0.001). Data are shown as the mean ± SEM or as individual data points (*n* = 5 per group for each sex).

To determine whether an initial effect of elevated serum ERFE on NEFA levels was blunted by tachyphylaxis after chronic exposure to ERFE, we measured serum NEFA concentrations in WT mice after the administration of a single dose of EPO. *Erfe* mRNA expression in the bone marrow and serum ERFE increased to levels comparable to those detected in mice chronically treated with EPO (Fig. 3A and B). However, serum NEFA levels were not different in mice that received acute EPO treatment compared with saline‐treated controls (Fig. 3C).

### The effect of ERFE on blood glucose homeostasis

To determine the influence of ERFE on blood glucose homeostasis we performed glucose tolerance testing (GTT) under basal conditions or after chronic EPO treatment in WT and *Erfe* ^*−/−*^ mice. After an overnight fast, baseline blood glucose levels trended to be lower in EPO‐treated compared with saline‐treated mice of the same genotype, as reported before (Foskett et al. 2011). This difference was statistically significant in male *Erfe* ^*−/−*^ and female WT mice but failed to reach significance in other groups. ERFE did not affect fasting blood glucose levels, as we observed no difference between WT and *Erfe* ^*−/−*^ mice under either basal or EPO‐stimulated conditions.

**Figure 4:**
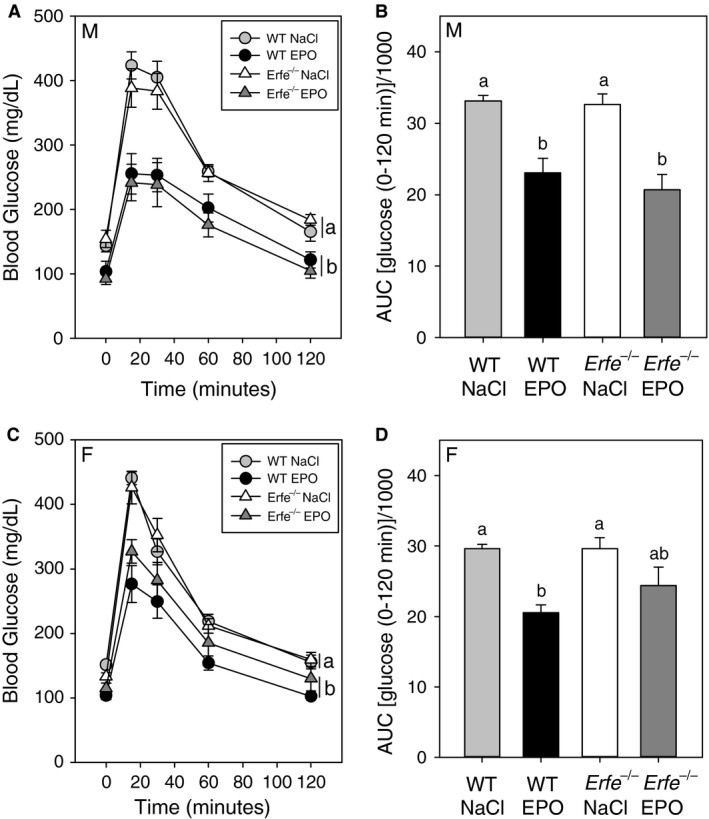
Erythroferrone does not affect glucose tolerance under basal conditions or after chronic treatment with EPO. Blood glucose levels during glucose tolerance testing in male (A) and female (C) mice. Curves that are not sharing a letter are significantly different as determined by two‐way repeated measures ANOVA (*P *<* *0.05). Area under the curve (AUC) analysis of blood glucose values for male (B) and female (D) mice during glucose tolerance testing. Groups of the same sex without a common alphabetical superscript differ significantly as determined by one‐way ANOVA (*P *<* *0.05). Data are shown as the mean ± SEM (*n* = 6–8 per group for each sex).

Analysis of blood glucose levels during GTT determined that, for both male and female mice, treatment with EPO altered glucose tolerance compared to saline treatment, but there was no effect of *Erfe* genotype (Fig. 4 A, C; two‐way repeated measures ANOVA followed by multiple comparisons testing). Area under the curve (AUC) analysis of blood glucose values during testing indicated that chronic EPO treatment improved glucose tolerance to a similar degree in both WT and *Erfe* ^*−/−*^ mice (Fig. 4B, D). AUC values during testing were significantly lower in male WT, male *Erfe* ^*−/−*^, and female WT mice after EPO treatment. In female *Erfe* ^*−/−*^ mice the difference in the AUC during testing in EPO‐treated mice compared with saline‐treated controls did not reach statistical significance.

### The effect of ERFE on insulin tolerance

To detect whether the lack of difference in glucose tolerance between WT and *Erfe* ^*−/−*^ mice was the result of glucose‐induced compensatory insulin secretion, we performed insulin tolerance testing (ITT) in mice used previously for GTT analysis. After a 6 h fast prior to testing, there was a trend toward lower blood glucose levels in mice chronically treated with EPO compared with saline‐treated mice, regardless of genotype. This difference was statistically significant in male mice but failed to reach significance in female mice.

**Figure 5:**
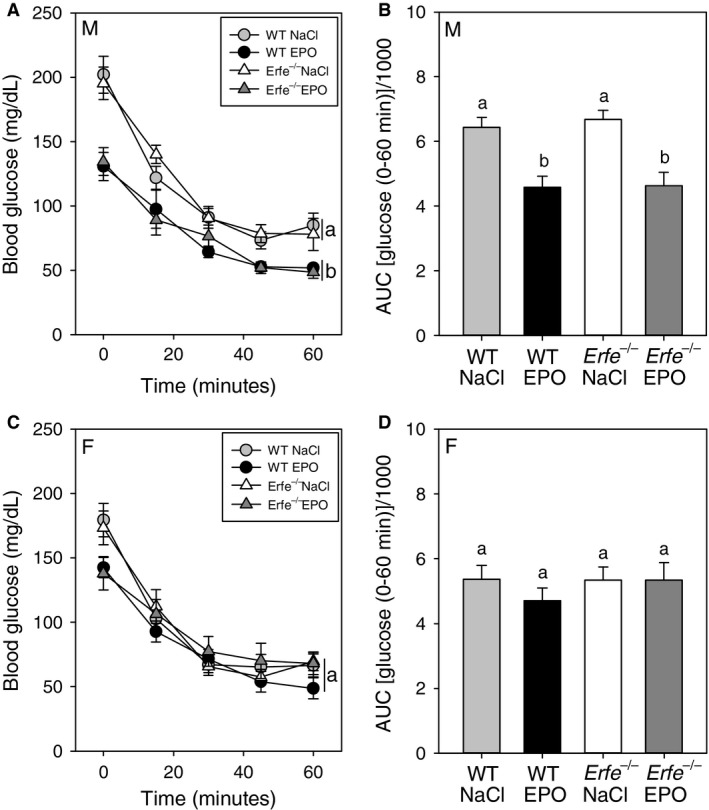
Erythroferrone does not affect insulin tolerance under basal conditions or after chronic treatment with EPO. Blood glucose levels during insulin tolerance testing in male (A) and female (C) WT and Erfe‐/‐mice chronically treated with either 200 U EPO or saline (NaCl). Curves not sharing a letter are significantly different as determined by two‐way repeated measures ANOVA (*P *<* *0.05). Area under the curve (AUC) analysis of blood glucose values for male (B) and female (D) mice during insulin tolerance testing. Groups without a common alphabetical superscript differ significantly from other groups of the same sex as determined by one‐way ANOVA (*P *<* *0.05). Data are shown as the mean ± SEM (*n* = 6–8 per group for each sex).

Analysis of blood glucose levels during insulin tolerance testing determined that in male mice EPO‐treated groups were significantly different from saline‐treated controls (Fig. 5A; two‐way repeated measures ANOVA followed by multiple comparisons testing). Groups differing in genotype, but not treatment, were not significantly different. AUC analysis of blood glucose levels during ITT also determined that blood glucose levels did not differ significantly between genotypes in either saline or EPO‐treated male mice (Fig. 5B). ITT of female mice failed to demonstrate any difference in insulin sensitivity between WT and *Erfe* ^*−/−*^ mice under basal or EPO‐stimulated conditions (Fig. 5C), as determined by two‐way repeated measures ANOVA followed by multiple comparisons testing. AUC values from EPO‐treated female mice during testing, from either genotype, were not different from saline‐treated controls (Fig. 5D).

### Contribution of ERFE to erythropoiesis in response to chronic EPO treatment

**Figure 6:**
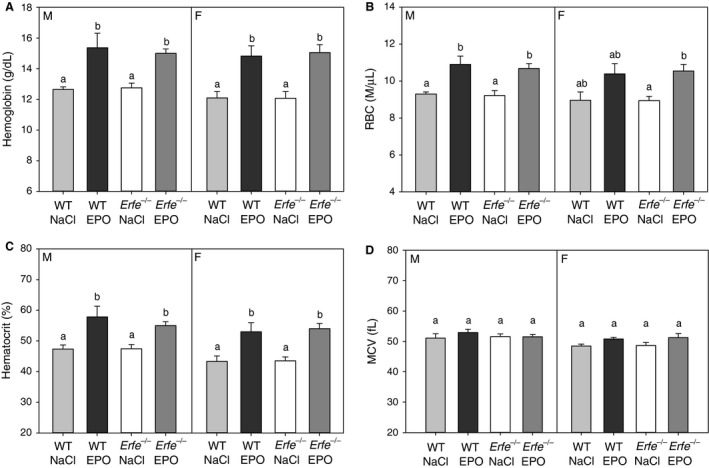
Stimulation of erythropoiesis by chronic erythropoietin treatment is preserved in mice lacking erythroferrone. Hemoglobin (A), red blood cell (B), hematocrit (C), and mean corpuscular volume (D) levels measured in male and female mice chronically treated with either 200 U EPO or saline (NaCl). Groups without a common alphabetical superscript differ significantly from other groups of the same sex as determined by one‐way ANOVA (*P *<* *0.05). Data are shown as the mean ± SEM (*n* = 5–6 per group for each sex).

No differences in hematological parameters were detected between WT and *Erfe* ^*−/−*^ mice during either basal, nonstimulated conditions or after chronic treatment with EPO (Fig. 6A–D). As expected, mice treated with EPO displayed elevated hemoglobin, red blood cell, and hematocrit levels compared with saline‐treated mice, although the trend toward increased red blood cell levels with EPO treatment did not reach statistical significance for female WT mice. Mean corpuscular volume was unaffected by either chronic EPO treatment or genotype.

**Figure 7:**
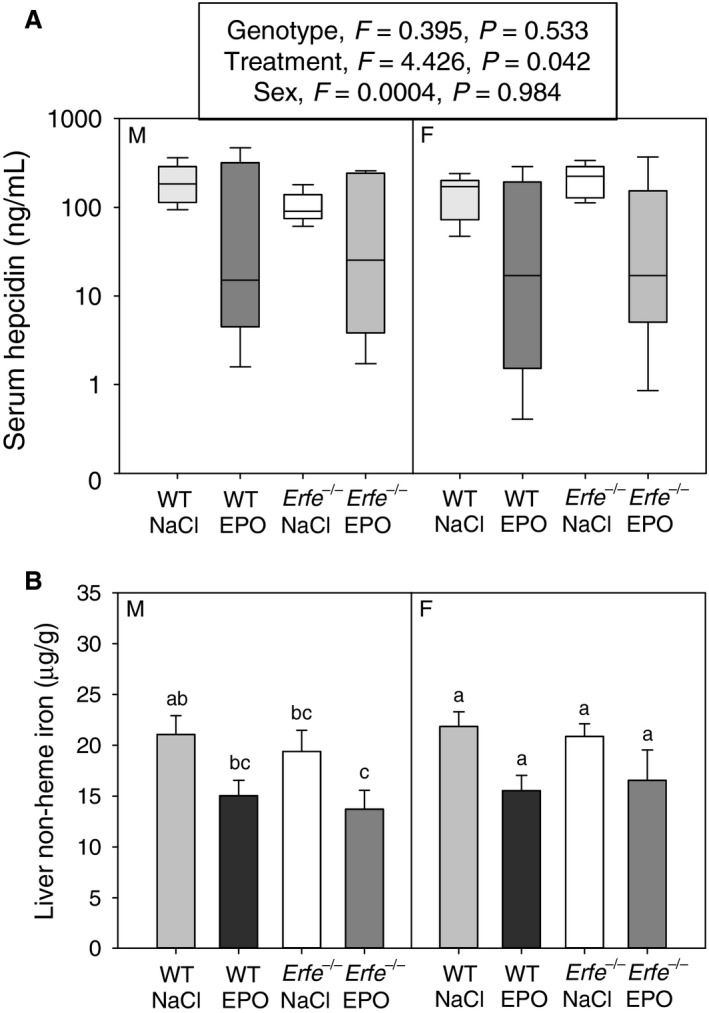
Liver iron content and hepcidin expression are not different between WT and *Erfe* ^*−/−*^ mice under basal conditions or after chronic EPO treatment. (A) Serum hepcidin concentrations in WT and *Erfe* ^*−/−*^ mice chronically treated with either EPO or saline (NaCl). Groups were analyzed by three‐way ANOVA (genotype, sex, treatment), and there was no significant interaction between factors (*P *<* *0.05). (B) Liver nonheme iron concentrations in WT and *Erfe* ^*−/−*^ mice chronically treated with either EPO or saline (NaCl). Groups without a common alphabetical superscript differ significantly from other groups of the same sex as determined by one‐way ANOVA (*P *<* *0.05). Data are shown as the mean ± SEM (*n* = 5–7 per group for each sex).

To determine if a lack of ERFE affects the mobilization of stored iron in response to chronic erythropoietic stimulation, we measured indices of iron status in WT and *Erfe* ^*−/−*^ mice treated with either EPO or saline. Serum hepcidin concentrations were significantly lower in mice treated chronically with EPO compared with those that received saline, as determined by three‐way ANOVA, but neither genotype nor sex had a detectable effect on serum hepcidin concentrations (Fig. 7A). In addition, no interaction between treatment, genotype, or sex was detected. In agreement with the lower hepcidin levels measured in EPO‐treated compared with saline‐treated mice of the same genotype, we detected a trend toward lower liver nonheme iron concentrations in mice that received EPO, although these differences did not reach statistical significance (Fig. 7B).

## Discussion

The erythroid hormone ERFE mediates acute hepcidin suppression in response to erythropoietic stimuli to match iron supply with erythropoietic demand (Kautz et al. 2014). However, a previous study suggested that ERFE plays a role in the regulation of blood lipid homeostasis (Seldin et al. 2012). Treatment with ERFE was reported to increase NEFA uptake by hepatocytes and adipocytes in vitro and decrease serum NEFA levels in mice in vivo (Seldin et al. 2012). As reducing circulating levels of NEFA improves insulin sensitivity and glucose tolerance (Santomauro et al. 1999), we aimed to test the possibility that beneficial effects of EPO treatment on glucose homeostasis (Allegra et al. 1996; Mak 1998; Katz et al. 2010; Foskett et al. 2011; Alnaeeli et al. 2014) are mediated by increased ERFE signaling. This study is the first to investigate the influence of physiologically relevant concentrations of ERFE on blood lipid and glucose homeostasis.

In contrast to the previous indication that ERFE may modulate serum lipid homeostasis, specifically serum NEFA levels, we detected no influence of ERFE on blood lipid parameters. Discrepancies between the previous and current study may be attributable to differences in the ERFE concentrations reached after exogenous ERFE administration as compared with those from endogenously produced ERFE. A transient decrease in serum NEFA was reported after the intraperitoneal administration of a supraphysiological dose, 5 *μ*g/gram body weight, of purified ERFE (Seldin et al. 2012). However, circulating levels of ERFE in mice range from below the threshold of detection, at baseline, to the low ng/mL range, in response to erythropoietic stimulation (Kautz et al. 2015). Therefore, changes observed in response to concentrations of ERFE exceeding those detected in vivo by orders of magnitude may not accurately represent the effect of ERFE in the context of physiological responses. It is also possible that at high doses recombinant ERFE acts as a noncognate ligand for receptors involved in the regulation of lipid homeostasis, accounting for the previously reported effect of ERFE on blood lipids.

As treatment with EPO simultaneously improves glucose tolerance and insulin sensitivity while stimulating the production of ERFE (Kautz et al. 2014, 2015), we investigated the possibility that improvements in blood glucose homeostasis in response to chronic EPO treatment are influenced by increased ERFE signaling. We found no effect of ERFE on the enhancement of glucose tolerance in response to prolonged treatment with EPO, as mice lacking ERFE demonstrated comparable glucose tolerance to that of WT mice after treatment with EPO. Determination of insulin sensitivity by ITT in this study also indicates that ERFE does not modulate insulin‐stimulated glucose clearance. Lower fasting blood glucose values in EPO‐treated mice hinder the determination of whether EPO treatment improved insulin sensitivity compared with saline‐treated controls in this study, because lower glucose concentrations in EPO‐treated mice may enhance counterregulatory responses (Jacobson et al. 2006) that could alleviate hypoglycemia during testing. However, the absence of ERFE did not affect insulin tolerance under basal or EPO stimulated conditions, as the response in *Erfe* ^*−/−*^ mice was similar to that observed in WT mice, suggesting that physiologic levels of ERFE do not affect insulin sensitivity, in agreement with the observed lack of effect on glucose tolerance. EPO may affect glucose metabolism by direct, EPO‐receptor mediated signaling, as mice lacking the EPO receptor in adipocytes develop glucose intolerance and insulin resistance (Wang et al. 2013).

In this study, erythropoiesis, hepcidin suppression, and depletion of iron stores in response to chronic EPO treatment were unaffected by the absence of ERFE. These findings suggest that ERFE, while acting as an acute regulator of iron homeostasis in response to erythropoietic stimulation, is dispensable for hepcidin suppression during prolonged moderate erythropoietic augmentation. One signal likely contributing to hepcidin suppression in this model is iron depletion induced by chronic EPO treatment (Fig. 7B). The idea that mechanisms independent of ERFE could contribute to the iron homeostatic response to increased erythropoiesis is also raised by the *Hbb* ^*th3/+*^ mouse, a model of *β* thalassemia intermedia (Yang et al. 1995), characterized by persistent anemia, increased EPO levels, and suppressed hepcidin expression, resulting in tissue iron accumulation (De Franceschi et al. 2006; Kautz et al. 2015). Although the hepcidin suppression is reversed by *Erfe* ablation, the iron overload phenotype of *Hbb* ^*th3/+*^ mice is only partially corrected (Kautz et al. 2015), suggesting either that hepcidin is still inappropriately low for the degree of iron overload or that a hepcidin‐resistant mechanism contributes to iron overload in these thalassemic mice. The demonstrable modulation of hepcidin by ERFE in *Hbb* ^*th3/+*^ mice, but not in this study, may be attributable to higher ERFE expression in *Hbb* ^*th3/+*^ mice (Kautz et al. 2015) compared with that measured after chronic EPO treatment. At moderately elevated levels of ERFE, as in this study, the influence of ERFE signaling on hepcidin suppression may be masked by other regulatory mechanisms but would still be discernable in models with more robust ERFE production, such as the *Hbb* ^*th3/+*^ mouse.

In conclusion, our data indicate that physiologic concentrations of ERFE do not affect blood lipid or glucose homeostasis and that the glucometabolic responses observed in response to chronic EPO treatment are ERFE‐independent. Moreover, chronic EPO therapy suppresses hepcidin by a mechanism that does not require ERFE. These findings add to the evidence that the primary physiologic function of ERFE is as a stress hormone in the setting of acute blood loss or other acute anemia where EPO‐stimulated ERFE suppresses hepcidin and thereby accelerates the supply of iron to compensatory erythropoiesis. As is the case with other hormones, ERFE may have noncanonical effects when its concentrations are unphysiologically increased to very high levels, as in many patients with *β*‐thalassemia or other disorders with ineffective erythropoiesis, or after the administration of exogenous ERFE.

## Conflicts of Interest

E.N. and T.G. are consultants and shareholders of Intrinsic LifeSciences and Silarus Therapeutics. The remaining authors declare no competing financial interests.
